# Endoscopic Management of Pancreatic Pseudocysts

**DOI:** 10.1155/DTE.1.29

**Published:** 1994

**Authors:** M. Dohmoto, K. D. Rupp

**Affiliations:** 1 Universität Klinik Rudolf Virchow/Fu-Berlin Chirurgische Klinik der Robert Rössle Klinik Lindenbergerweg 80 Berlin 13122 Germany; 2 Chirurgische Klinik der Ruhr-Universität Bochum Hölkeskampring 40 Herne 44625 Germany; 3 Rosental 24 Hattingen 45525 Germany

## Abstract

Recently, endoscopic interventional procedures were introduced for nonsurgical therapy of symptomatic pancreas pseudocysts. We reported 25 patients treated by endoscopic retrograde pancreas drainage (ERPD), endoscopic cystogastrostomy (ECG), or endosopic cystoduodenostomy (ECD).

ERPD was performed in 9 patients by placement of a 5 Fr. or 7 Fr. endoprosthesis transpapillary into the cyst or the main pancreatic duct. ECG was carried out in 10 cases, in 7 of these, a double pigtail catheter was additionally inserted. Three patients suffering from pseudocysts of the pancreas head were treated by ECD. In a further 3 cases, ERPD and ECG were combined.

All patients reported a dramatic reduction of pain with a simultaneous increase of appetite and body weight. The drainage tubes were removed after disappearance of symptoms, and abnormal clinical and endoscopic findings within 2 to 12 months. In 4 cases, a recurrence of the cyst was found 10 and 22 months later, in 3 cases the endoprostheses had to be renewed because of catheter occlusion or dislocation. 2 patient underwent surgical treatment after insufficient endoscopic drainage due to haemorrhage or recurrence.

Endoscopic treatment of pancreatic pseudocysts yielded good results with low rates of recurrence and complications. According to our experiences we think endoscopic interventional techniques will oust surgery from its present dominant position in the next years.


*Diagnostic and Therapeutic Endoscopy*, Vol. 1, pp. 29–35
Reprints available directly from the publisher
Photocopying permitted by license only

© 1994 Harwood Academic Publishers GmbH
Printed in Malaysia

### Endoscopic Management of Pancreatic Pseudocysts

M. DOHMOTO[1] and K. D. RUPP[2]

[1]*Universität Klinik Rudolf Virchow/Fu-Berlin, Chirurgische Klinik der Robert Rössle Klinik, Lindenbergerweg 80, 13122 Berlin Germany,*
[2]*Chirurgische Klinik der Ruhr-Universität Bochum Hölkeskampring 40, 44625 Herne, Germany*

*(Received September 30, 1993; in final form February 10, 1994)*

Recently, endoscopic interventional procedures were introduced for nonsurgical therapy of symptomatic pancreas pseudocysts. We reported 25 patients treated by endoscopic retrograde pancreas drainage (ERPD), endoscopic cystogastrostomy (ECG), or endosopic cystoduodenostomy (ECD).

ERPD was performed in 9 patients by placement of a 5 Fr. or 7 Fr. endoprosthesis transpapillary into the cyst or the main pancreatic duct. ECG was carried out in 10 cases, in 7 of these, a double pigtail catheter was additionally inserted. Three patients suffering from pseudocysts of the pancreas head were treated by ECD. In a further 3 cases, ERPD and ECG were combined.

All patients reported a dramatic reduction of pain with a simultaneous increase of appetite and body weight. The drainage tubes were removed after disappearance of symptoms, and abnormal clinical and endoscopic findings within 2 to 12 months. In 4 cases, a recurrence of the cyst was found 10 and 22 months later, in 3 cases the endoprostheses had to be renewed because of catheter occlusion or dislocation. 2 patient underwent surgical treatment after insufficient endoscopic drainage due to haemorrhage or recurrence.

Endoscopic treatment of pancreatic pseudocysts yielded good results with low rates of recurrence and complications. According to our experiences we think endoscopic interventional techniques will oust surgery from its present dominant position in the next years.

**KEY WORDS:** pancreatic pseudocysts, endoscopic drainage of pancreatic pseudocyst

### INTRODUCTION

Pancreatic pseudocysts can occur in 20–50% of chronic pancreatitis cases (Sarles *et al.*, 1979; Malfertheiner *et al.*, 1988). In contrast to acute pancreatitis, pancreatic pseudocysts due to chronic pancreatitis do not usually disappear spontaneously. Small asymptomatic cysts do not need any treatment other than regular observation. Only large asymptomatic cysts measuring more than 5 cm in diameter should be drained because of the high risk of developing complications. All cysts causing symptoms such as pain, loss of appetite and weight, or complications such as bleeding, infection, or jaundice due to compression of the common bile duct also require drainage (Frey, 1981; Mullins *et al.*, 1988; Sulkowski *et al.*, 1991).

For many years, surgery was the only type of treatment for pancreatic pseudocysts. In addition to surgical techniques, radiological and sonographic method of external and internal drainage have recently been introduced. Corresponding to the rapid development of interventional endoscopy with the first placement of a biliary drainage by Soehendra in 1979, today there are also several endoscopic procedures in the treatment of pancreatic pseudocysts. In the present paper, we report our experiences with the transpapillary endoscopic retrograde pancreatic drainage (ERPD), the endoscopic cystogastrostomy (ECG), and the endoscopic cystoduodeno-stomy (ECD). The aim of these endoscopic methods is to drain the stagnant pancreatic pseudocyst contents through the prosthesis or the cystostomy into the digestive tract using the same principle as the surgical construction of an internal fistula (Dohmoto *et al.*, 1992). The purposes are to relieve pain, prevent complications, restore the exocrine function, and to stop the mostly underlying inflammatory disease for preservation of the endocrine function (Soehendra *et al.*, 1986).

Address for correspondence: M. Dohmoto, M. D., Rosental 24, 45525 Hattingen, Germany.

### MATERIALS

In a period of 66 months, 25 patients suffering sypmptomatic pancreatic pseudocysts were treated by endoscopic drainage. In 19 cases, the cysts were due to chronic pancreatitis caused by alcoholism in 18 patients. One cyst developed after iatrogenic intraoperative damage to the pancreas, and 1 purulent cyst was due to acute biliary pancreatitis. In 6 cases, the etiology was unknown. Twenty patients complained of severe continuous or relapsing pain; 12 reported a loss of appetite and weight. In 7 cases, signs of sepsis were observed. The female:male ratio was 13:12. The age range was from 18 to 89 years (average age, 49.7 years) (Table 1).

**Table 1** Endoscopic Drainage of Pancreatic Pseudocysts (n=25)

| | |
|---|---|
| Etiology | |
| Chronic pancreatitis | 19 |
| Alcoholism | 18 |
| Unknown etiology | 6 |
| Iatrogenic pseudocyst | 1 |
| Symptoms | |
| Continuous or relapsing pain | 20 |
| Inappetence | 12 |
| Sepsis | 7 |

male: 13, female: 12, Age: 49.7 (18–89) yrs.

### METHODS

Before undergoing endoscopic drainage of pancreatic pseudocysts, all patients particularly those with chronic pancreatitis should be carefully examined for signs of pancreatic carcinoma. The diagnostic procedure includes sonography, computer-tomography, ERCP, laboratory findings including tumor markers, and if necessary, cytology. The instruments and pre-procedural work-up of patients for endoscopic drainage of pancreatic cysts are similar to that for endoscopic biliary drainage using an operative duodenoscope (ERPD and ECD) or gastroscope (ECG) with working channels of 2.8–3.7 mm.

ERPD is typically done for single or multiple cysts communicating with the main pancreatic duct (Fig. 1a). Most of these pseudocysts develop from ductal distension proximal to a ductal stenosis. In ERPD cases, of the stenotic site of the pancreatic duct is dilated along a guidewire under x-ray monitoring. Afterwards, a 5 or 7 Fr. endoprosthesis is inserted through the dilated stenosis into the pancreatic tail (Fig. 1b).

ECG is performed in cases of pancreatic pseudocysts with direct contact to the stomach (Fig. 2a) but without communication to the pancreatic main duct. These cysts are located mostly in the pancreatic corpus. Large cysts can be identified as a prominent bulging of the posterior gastric wall (Fig. 3a), smaller ones only by means of endosonography. The cysts are incised with round tip basket forceps using coagulation current. Through a small

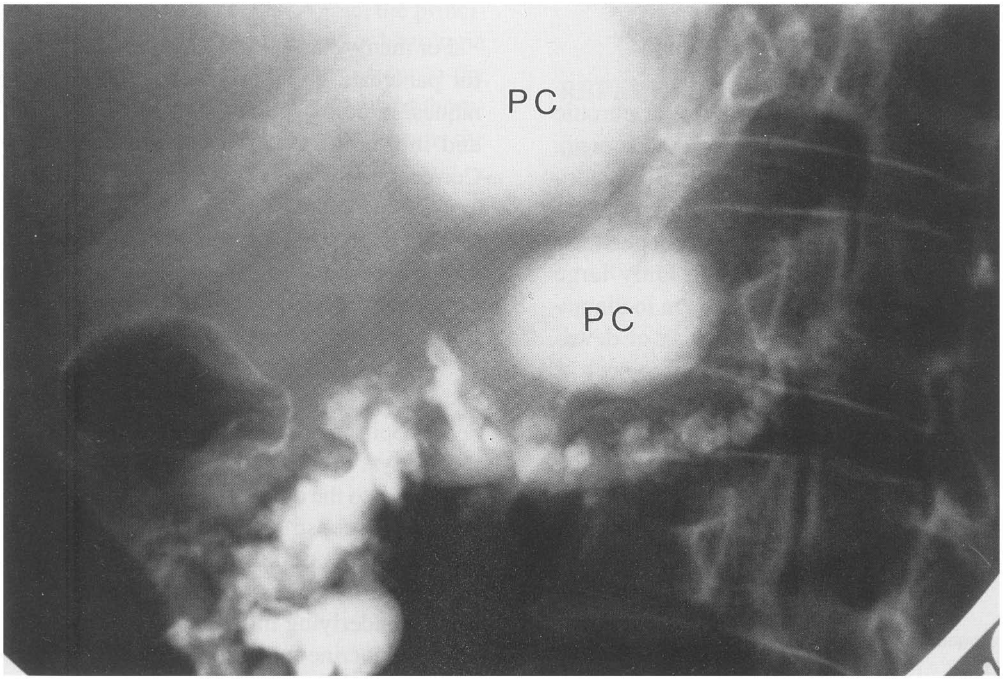

**Figure 1a** Endoscopic retrograde pancreatic drainage (ERPD). 1a. ERP shows advanced chronic pancreatitis with large pancreatic pseudocysts communicating with the dilated pancreatic duct.

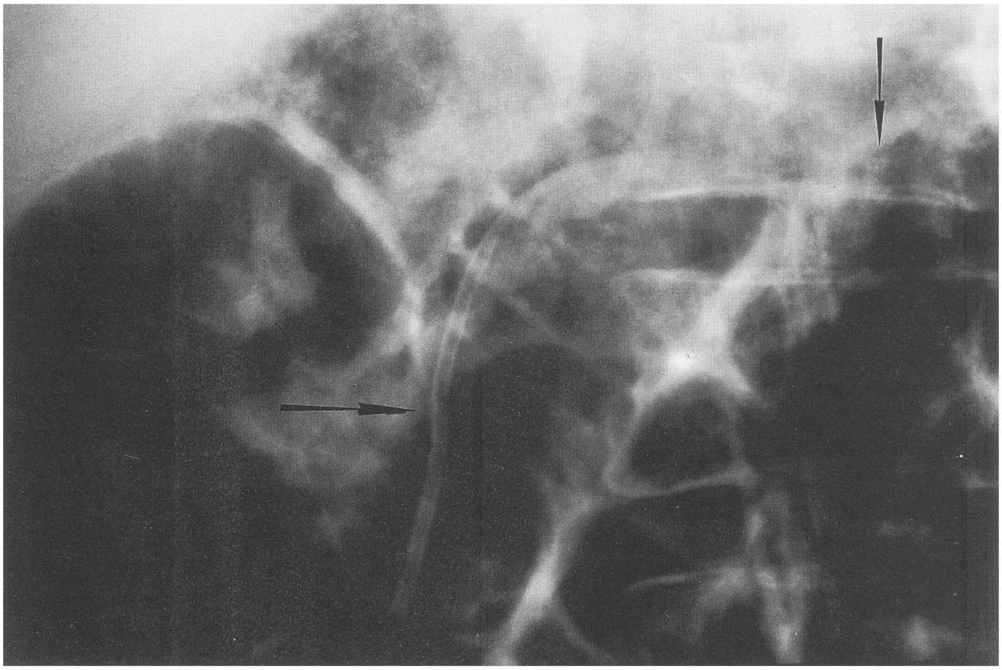

**Figure 1b** Endoscopic retrograde pancreatic drainage (ERPD). 1b. A 7 Fr. prosthesis is inserted into the pancreatic duct (ERPD).

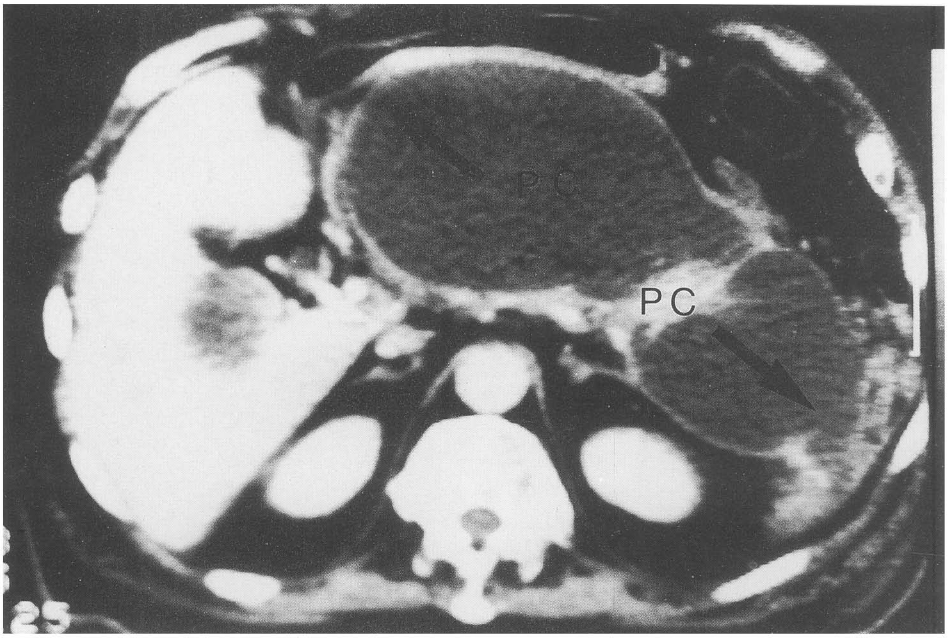

**Figure 2a** Trans gastrale cystostomy (ECG). 2a. CT-scan shows a largely retrogastral pseudocyst.

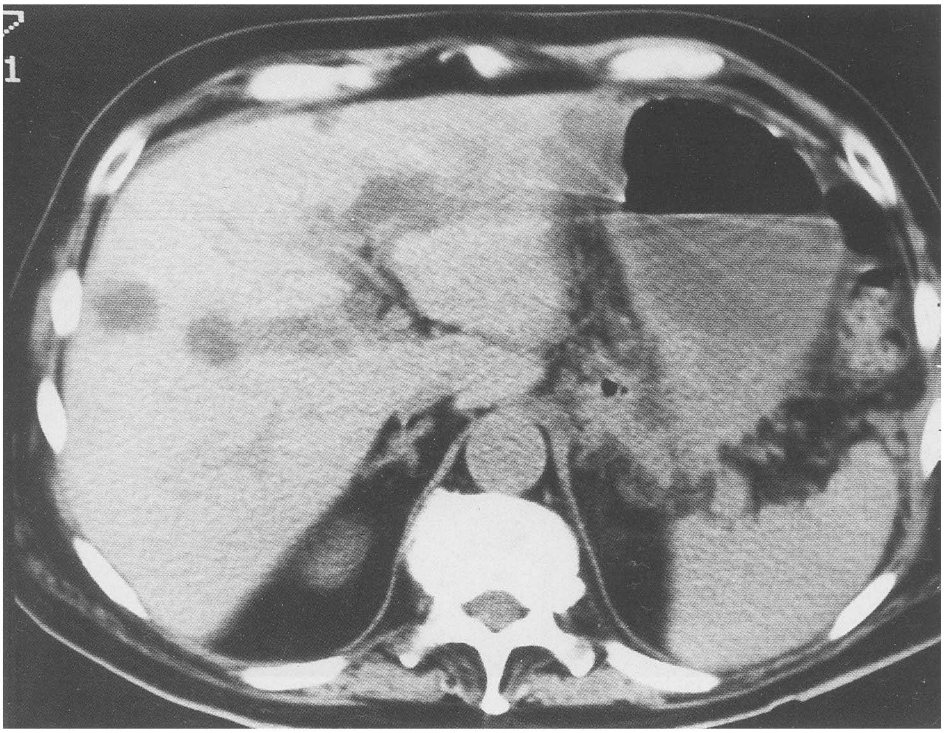

**Figure 2b** Trans gastrale cystostomy (ECG). 2b. 5 weeks later the cyst completely disappeared.

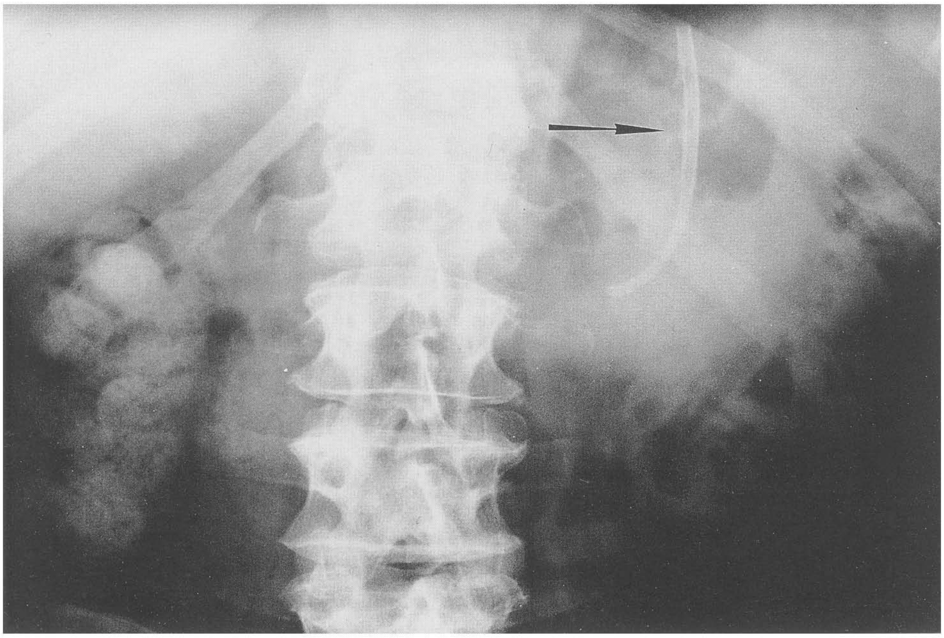

**Figure 2c** Trans gastrale cystostomy (ECG). 2c. X-ray control of transgastral pancreatic prosthesis (ECG).

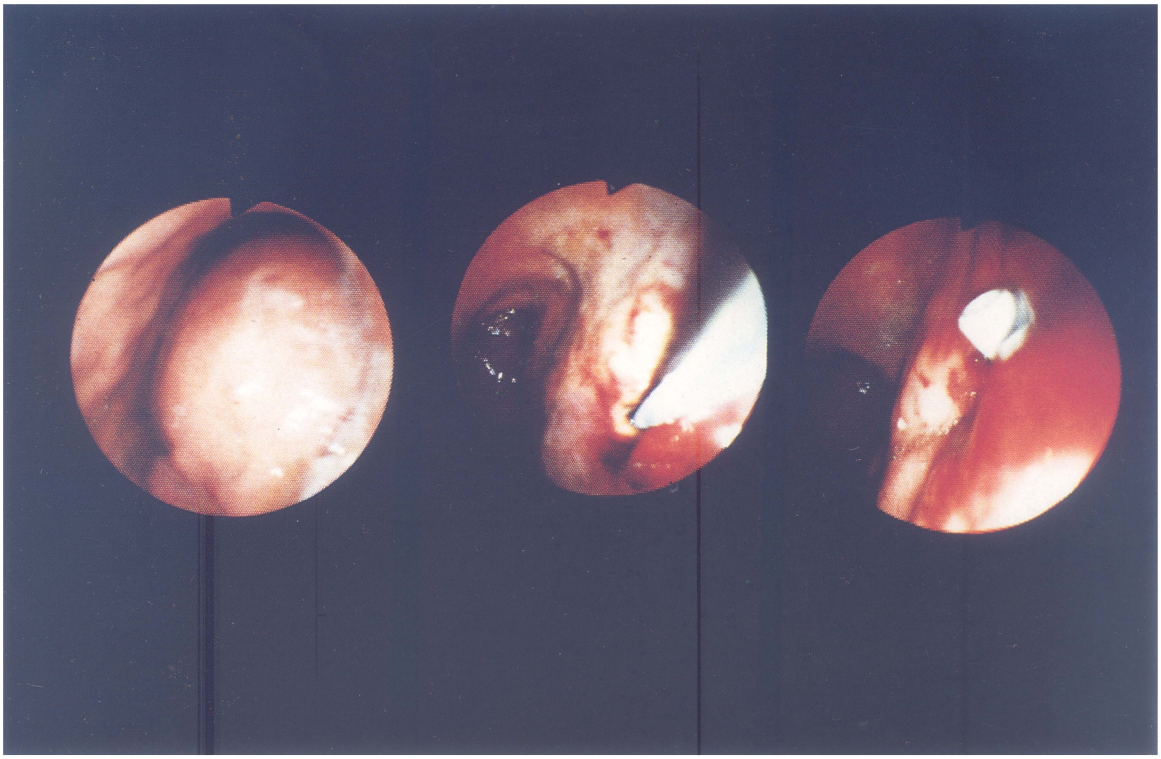

a b c

**Figure 3a–c** Endoscopic findings of ECG. 3a. A large retrogastric pseudocyst is bulging the posterior stomach wall. 3b. After transgastral incision of the pseudocyst, the prosthesis is inserted into the pseudocyst. 3c. Final position of Fr. 7 prosthesis.

incision of 3 mm, the basket forceps were inserted and contrast liquid instilled to image the cyst radiologically. Subsequently, the cystotomy is enlarged to 5 mm with a short papillotome (Fig. 3b), and after aspiration of the cyst contents, one or more 7 Fr. pigtail endoprostheses were inserted (Figs. 2c and 3c). ECD is indicated for cysts of the pancreatic head impressing the duodenum. The incision and drainage is similar to the ECG procedure.

ERPD and ECG or ECD are combined in cases of multiple cysts if one cyst cannot be drained due to lack of communication with the pancreatic duct (Fig. 4).

In all procedures, antibiotics, mucosal protectiva, and $H_2$-blockers were administered. After some days of stationary observation, the patient was discharged. During the following months, regular clinical, hematological, and endoscopic examinations should be carried out on an ambulatory basis. The drainage tubes should be removed after disappearance of symptoms and resolving of the cyst.

### RESULTS

Over a period of 66 months, 25 of 29 patients with pancreatic pseudocysts requiring therapy were successfully treated by endoscopic drainage procedures. In the remaining 4 cases, transpapillary drainage did not succeed because of massive calcifications of the pancreas or tortuously convoluted stenotic pancreatic ducts. In 9 patients with cysts communicating with the main pancreatic duct, ERPD was carried out despite marked calcifications seen in 5 of these. ECG was done 9 times, in 7 of these cases with additional insertion of pigtail endoprostheses. ECD was performed 3 times, in one case of purulent pseudocyst with additional insertion of a naso-cystic catheter for continous lavage of the cyst cavity for a few days. ERPD and ECG were combined in 3 patients (Table 2).

After endoscopic cyst drainage, mitigation of pain and postprandial epigastralgia was observed by all patients. Due to increasing appetite, patients in poor nutritional condition gained up to 16 kg of weight. After the disappearance of symptoms and abnormal endoscopic and clinical findings within a period of 2 to 12 months, the drainage tubes were removed (Fig. 2b).

In 4 cases, a recurrence of the cyst was found 10 and 22 months later, in 3 cases the endoprostheses had to be renewed because of catheter occlusion or dislocation. One patient underwent surgical treatment after insufficient endoscopic drainage. Failure was caused by a bleeding after ECG filling the cavity of the cyst with coagulated blood.

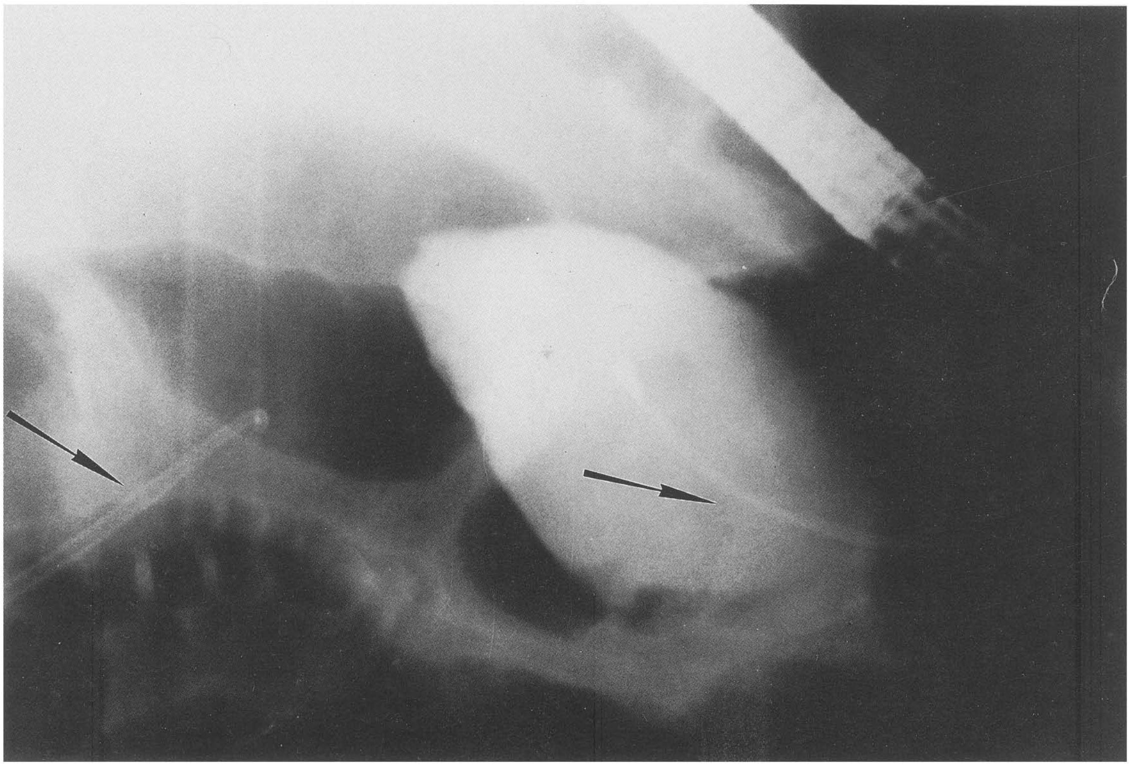

**Figure 4** Combination drainage with transpapillary and transgastral catheters.

**Table 2** Endoscopic Drainage of Pancreatic Pseudocysts

| *ERCP findings (n=25)* | *ERPD* | *ECG* | *ECD* | *ERPD+ECG* |
|---|---|---|---|---|
| Pseudocyst in communication to pancreatic duct | 9 | | | 3* |
| Pseudocyst without communication to pancreatic duct | | 10 | 3 | 3* |
| Location of pseudocyst | | | | |
| Pancreatic head | 2 | | 3 | |
| Pancreatic corpus | 4 | 8 | | |
| Pancreatic tail | 2 | 1 | | |
| Multiple | 1 | 1 | | 3* |

* Same patients.

In order to achieve cystointestinal drainage, cystojejunostomy was surgically done after removal of the haematoma. Cystojejunostomy also had to be performed in 1 case due to recurrence (Table 3).

### DISCUSSION

Large pancreatic pseudocysts in particular were related with regard to complications such as bleeding, rupture, abscess, or fistula in up to 55% of cases (Bradly 1984, Wade 1985, Zirngibl *et al.*, 1983). These large cysts over 5 cm in diameter, and every cyst causing symptoms require treatment. For many years, surgery was the only available therapy. Operative cystojejunostomy is attended with a rate of complication of 14 to 41%, and a mortality of 3 to 9%. Recurrence of cysts is observed in 0 to 7% of case. In resective surgery of the pancreas, morbidity and mortality is much higher (Freeny *et al.*, 1988; Heyder *et al.*, 1988; Hollender *et al.*, 1988, Nguyen *et al.*, 1991; Sankaran *et al.*, 1975, Scatney *et al.*, 1979; Spinelli *et al.*, 1988; Stanley *et al.*, 1976).

With the introduction of sonography, compute tomography, and endoscopy, a number of interventional procedures were presented. Percutaneous puncture of the cyst under sonographic or radiological guidance is a method to practice easily. In most cases, however, repeated punctures are necessary due to high rates of recurrence. These

**Table 3** Outcome of Endoscopic Drainage of Pancreatic Pseudocysts (n=25)

| *Follow-up (n)* | *ERPD 9* | *ERPD+ECG 3* | *ECG 10* | *ECD 3* |
|---|---|---|---|---|
| Recurrence | 1 | 1 | 2 | |
| Bleeding | | 1 | | |
| Catheter occlusion | 2 | | | |
| Cystojejunostomy | | 2 | | |
| Endoscopic redrain | 1 | | 2 | |
| Dead | 1* | | | |

*Myocardiac insufficiency.

circumstances make the method rather uncomfortable for the patient. Another choice of therapy is continous percutaneous drainage, but there is a rather high risk of chronic pancreatico-cutaneous fistula and infections. The rate of success amounts to 80% (Frey 1981; Grosso *et al.*, 1989; Hancke *et al.*, 1985, McConnell *et al.*, 1982).

There also are many endoscopic procedures like endoscopic guided percutaneous drainage or endoscopic placement of a naso-cystic tube. The best success with a average of 95% show internal drainages like ECG, ECD and ERPD. Beyond this they are better accepted by the patients because there are no tubes or bags hanging outside the body. Rate of complication is about 10%, mortality ranged from 0 to 5.5% and recurrence of the cyst is observed in 9 to 19% (*23–28*) (Cremer *et al.*, 1989, Grimm *et al.*, 1989; Huigbregtse *et al.*, 1989; Kozarek, 1985 and 1990, Malfertheiner *et al.*, 1991, Sahel, 1991).

Our own experiences with endoscopic internal drainage of pancreatic pseudocysts are similar to the excellent results that have been published recently. According to the high rates of success in combination with low morbidity and mortality, these endoscopic procedures should be the therapy of first choice in the treatment of pancreatic pseudocysts, particularly in high-risk patients. Further advantages in comparison to surgery are the shorter stationary stay of the patient and the lower costs.

If endoscopic treatment fails or is technically impossible due to lack of communication or contact of the cyst with the pancreatic main duct or the gastric and duodenal wall, surgical therapy should be carried out. From our point of view, there is no indication for external drainage because of the discomfort for the patient causing a low acceptance. The only exception may be the general or local inoperable patient.

### REFERENCES

Bradly E. L.: Cystoduodenostomy-new perspectives. *Ann Surg* 1984;200:698–702

Cremer M., Deviere J., Engelholm L.: Endoscopic management of cysts and pseudo-cysts in chronic pancreatitis: longterm follow up after 7 years of experience. *Gastrointest Endoscopy* 1989;35:1–9

Dohmoto M., Rupp K. D.: Endoscopic drainage of pancreatic pseudocysts. *Surg Endosc* 1992;6:118–124

Freeny P. C., Lewiss G. P., Traverso L. W. *et al.*: Infected pancreatic fluid collections: percutaneous catheter drainage. *Radiology* 1988;167:435–441

Frey C. F.: Pancreatic pseudocyst operative strategy. *Ann Surg* 1978;188:652–662

Frey C. F.: Role of subtotal pancreatectomy and pancreaticojejunostomy in chronic pancreatitis. *J Surg Res* 1981 Nov.;31(5):361–370

Grimm H., Meyer W. H., Nam V. Ch. *et al.*: New modalities for treating chronic pancreatitis. *Endoscopy* 1989;21:70–74

Grosso M., Gandini G., Cassinis M. S. *et al.*: Percutaneous treatment (including pseudo-cystogastrostomy) of 74 pancreatic pseudocysts. *Radiology* 1989;173:493–497

Hanecke S., Henriksen F. W.: Percutaneous pancreatic cystogastrostomy guided by ultrasound scanning and gastroscopy. *Brit J Surg* 1985;72:916–917

Heyder N., Flügel H., Domschcke W.: Catheter drainage of pancreatic pseudocysts into the stomach. *Endoscopy* 1988;20:75–77

Hollender L. F., Peiper H. J. (Hrsg.): Pankreaschirurgie. Springer Verlag, Berlin, 1988

Huigbregtse K., Schneider B., Vrij A. A. *et al.*: Endoscopic pancreatic drainage in chronic pancreatitis. *Gastrointest Endoscopy* 1989;34:9–15

Kozarek R. A., Ball T. J., Patterson D. J.: Endoscopic transpapillary therapy for disrupted pancreatic duct and pseudocyst. *Gastrointest Endoscopy* 1990;36:203–207

Malfertheiner P., Büchler M.: Indications for endoscopic or surgical therapy in chronic pancreatitis. *Endoscopy* 1991;23:185–190

Malfertheiner P., Büchler M., Stanescu A., *et al.*: Pancreatic pseudocysts in chronic pancreatitis diagnosis and clinical picture. *Digestion* 1988;40:100

McConnell D. B., Gregory J. R., Sasaki T. M. *et al.*: Pancreatic pseudocyst. *Am J Surg* 1982;143:599–602

Mullins R. J., Malagoni M. A., Bergamini T. M. *et al.*: Contro-versies in the management of pancreatic pseudocysts. *Am J Surg* 1988;155:165–172

Nguyen B. L. T., Thompson J. S., Edny J. A., *et al.*: Influence of the etiology of pancreatitis on the natural history of pancreatic pseudocysts. *Am j Surg* Dez. 1991;162:527–531

Sahel J.: Endoscopic drainage of pancreatic cysts. *Endoscopy* 1991;23:181–184

Sankaran S., Walt A. J.: The natural and unnatural history of pancreatic pseudocysts. *Brit J Surg* 1975;62:37–44

Sarles J. C., Sahel J., Sarles H.: Cysts and pseudocysts of the pancreas. In: Howart H. T., Sarles H. (eds.): *The Exocrine Pancreas* Saunders, 1979

Scatney C. H., Lillehei R. L.: Surgical treatment of pancreatic cysts. *Ann. Surg.* 1979;189:386–390

Soehendra N., Grimm H., Schreiber H. W.: Endoscopische transpapilläre drainage des ductus wirsungianus bei der chronischen pankreatitis. *Dtsch. Med. Wschr.* 1986;111:727–731

Spinelli P., Meroni E., Prada A.: Endoscopic treatment of a pancreatic pseudocyst by nasocystic tube. *Endoscopy* 1988;20:27–29

Stanley S. C., Frey C. F., Miller T. A.: Major arterial hemorrhage: A complication of pancreatic pseudocysts and chronic pancreatitis. *Arch Surg.* 1976;111:435–439

Sulkowski U., Meyer J.: Erkrankungen des pankreas. *Diagnostik und Therapie Deutscher Ärzte-Verlag*, Köln, 1991;110–122

Wade J. W.: 25 years experience with pancreatic pseudocysts. *Am J Surg* 1985;149:705–708

Zirngibl H., Gebhardt C., Faβbender D.: Drainagebehandlung von pancreas-pseudo-cysten. *Langenbeck Arch Chir.* 1983;360:29–41

